# Phenformin suppresses angiogenesis through the regulation of exosomal microRNA-1246 and microRNA-205 levels derived from oral squamous cell carcinoma cells

**DOI:** 10.3389/fonc.2022.943477

**Published:** 2022-09-08

**Authors:** Dexuan Zhuang, Shuangshuang Wang, Guanyi Liu, Panpan Liu, Huiting Deng, Jianfeng Sun, Chang Liu, Xue Leng, Qun Zhang, Fuxiang Bai, Jun Mi, Xunwei Wu

**Affiliations:** ^1^ Shandong Key Laboratory of Oral Tissue Regeneration, Shandong Engineering Laboratory for Dental Materials and Oral Tissue Regeneration, Department of Tissue Engineering and Regeneration, School and Hospital of Stomatology, Cheeloo College of Medicine, Shandong University, Jinan, China; ^2^ Shandong Engineering Laboratory for Dental Materials and Oral Tissue Regeneration, Department of Orthodontics, School and Hospital of Stomatology, Cheeloo College of Medicine, Shandong University, Jinan, China; ^3^ Shandong Key Laboratory of Oral Tissue Regeneration, Shandong Engineering Laboratory for Dental Materials and Oral Tissue Regeneration, Department of Pediatrics Dentistry, Department of Preventive Dentistry, School and Hospital of Stomatology, Cheeloo College of Medicine, Shandong University, Jinan, China; ^4^ Engineering Laboratory for Biomaterials and Tissue Regeneration, Ningbo Stomatology Hospital, Savaid Stomatology School, Hangzhou Medical College, Ningbo, China; ^5^ Suzhou Research Institute, Shandong University, Suzhou, China

**Keywords:** exosomes, oral squamous cell carcinoma cells, angiogenesis, miR-1246, miR-205

## Abstract

Exosomes secreted by cancer cells are important components in the tumor microenvironment, enabling cancer cells to communicate with each other and with noncancerous cells to play important roles in tumor progression and metastasis. Phenformin, a biguanide antidiabetic drug, has been reported to have a strong antitumor function in multiple types of cancer cells, however little research has been reported about whether phenformin can regulate the secretion of exosomes by cancer cells to regulate the tumor microenvironment and contribute to its antitumor function. Here we found that exosomes (Phen-Exo) derived from phenformin-treated oral squamous cell carcinoma (OSCC) cells significantly suppress the proliferation, migration and tube formation of human umbilical vein endothelial cells (HUVECs) *in vitro*. The inhibition of angiogenesis by Phen-Exo was verified *in vivo* by matrigel plug angiogenesis assays and by chick chorioallantoic membrane assays. Mechanistically, we discovered that the expression of microRNA-1246 (miR-1246) and microRNA-205 (miR-205) was significantly increased in exosomes secreted by OSCC cells treated with phenformin, while high expression levels of miR-1246 or miR-205 in vascular endothelial cells inhibited their angiogenic effects and decreased expression of the angiogenic factor VEGFA. In conclusion, these results reveal that phenformin can inhibit angiogenesis by regulating the levels of miR-1246 and miR-205 in exosomes secreted by OSCC cells, suggesting that phenformin has the potential to alter the tumor microenvironment to antagonize the growth of OSCCs, which provides a theoretical basis for developing new strategies to treat OSCCs in the future.

## Introduction

Oral squamous cell carcinomas (OSCCs) are the most common type of malignant oral tumor with a high incidence that easily metastasizes to distant sites (1). OSCCs have a poor overall survival rate and a poor prognosis, which seriously affects patient survival (2). Although there are many treatment methods for OSCCs, including surgery, radiotherapy, chemotherapy, immunotherapy, nutritional therapy, cryotherapy, thermotherapy and Chinese herbal medicines, the treatment of OSCCs is still a major challenge in clinical practice (3). Moreover, previous studies have mainly focused on the relationship between genetic changes and the development of OSCCs. With further study, the discovery of the critical importance of the tumor microenvironment in the development and progression of OSCCs may provide new ideas for candidate diagnostic biomarkers and promising therapeutic targets for OSCCs (4–6).

Exosomes are important components of the tumor microenvironment, and many types of cells secrete extracellular vesicles under normal and pathological conditions, which is an important mediator of intercellular communication (7, 8). Exosomes secreted by tumor cells can carry a variety of genetic materials from tumor cells, such as microRNAs (miRNAs), DNA, proteins, etc. Among them, miRNAs in exosomes play a crucial role in regulating tumor growth, metastasis, angiogenesis, immune escape, drug resistance, etc. (9). Therefore, the study of factors that act as anticancer agents by regulating exosomal composition and their secretion from tumor cells is a hot topic of current research. Metformin, a drug commonly used in the treatment of diabetes, has been found to have a wide range of anticancer effects including breast cancer, prostate cancer and colon cancer (10–13). Recent studies have shown that metformin can affect the growth of tumor cells by influencing the synthesis and secretion of exosomes in tumor cells (14, 15). However, other recent studies have found that another biguanide hypoglycemic agent, phenformin, has significantly better anticancer effects than metformin (16, 17) and phenformin was also found to potentially inhibit angiogenesis by changing the tumor microenvironment, although the exact mechanism involved remains to be investigated (18).

Previous studies have focused on the direct role of phenformin in cancer cells and only a few studies have examined the effect of phenformin in the tumor microenvironment, and the association of phenformin with OSCCs has not been studied so far. Thus, the aim of this study was to investigate whether phenformin can affect the tumor microenvironment by regulating the secretion of exosomes from OSCC cells to affect the angiogenesis of endothelial cells and to study the potential underlying molecular mechanisms involved.

## Materials and methods

### Reagents

A phenformin (MedChemExpress, USA) stock solution (200 mM) was prepared by dissolving the compound in phosphate-buffered saline (PBS, Thermo Fisher Scientific, Cat. 10,010,049, USA), which was diluted with growth medium to the desired concentrations as indicated in the Figures.

### Cell lines and culture conditions

Human CAL 27 and SCC-9 OSCC cell lines were obtained from the American Type Culture Collection (ATCC, USA). Human umbilical vein endothelial cells (HUVECs) were from Shanghai Zhong Qiao Xin Zhou Biotechnology (Shanghai, China). CAL 27 cells were maintained in Dulbecco’s modified Eagle’s medium (DMEM) (Gibco, Cat. A4,192,101, USA) containing 10% fetal bovine serum (FBS) (Gibco, Cat. 16,000,044) and 1% penicillin-streptomycin (Thermo Fisher Scientific, Cat. 10,378,016). SCC-9 cells were cultured in DMEM:F-12 Medium (DMEM-F12) (ATCC, Cat. 30-2006) supplemented with 10% FBS, 1% penicillin-streptomycin and 400 ng/mL hydrocortisone (Solarbio, Cat. G8450, China). HUVECs were maintained in Endothelial Cell Medium (Shanghai Zhong Qiao Xin Zhou Biotechnology, Cat. ZQ-1304) containing 5% FBS (Shanghai Zhong Qiao Xin Zhou Biotechnology, Cat. ZQ-1304), 1% Penicillin/Streptomycin Solution (Shanghai Zhong Qiao Xin Zhou Biotechnology, Cat. ZQ-1304) and 1% Endothelial Cell Growth Supplement (Shanghai Zhong Qiao Xin Zhou Biotechnology, Cat. ZQ-1304).

### Isolation, purification and characterization of cell-derived exosomes

CAL 27 and SCC-9 cells were treated with PBS (PBS-Exo group) or with 1 mM phenformin (Phen-Exo group) for 48 h, when the cell density reached around 60%. The cell supernatants (conditioned medium, CM) were collected and placed in a freezer at -80°C for storage, or were directly isolated and purified using an exosome concentration solution (ECS) kit (ECS reagent, Umibio, Cat. UR52121, China). The conditioned medium and ECS reagent were shaken and mixed, then left to stand at 4°C for 2 h. The mixture was then centrifuged at 10,000 g for 60 min at 4°C, and the supernatant was discarded. The precipitate was then resuspended in PBS, centrifuged at 12,000 g for 2 min at 4°C and at 3,000 g for 10 min at 4°C in a centrifugation tube. Next, the post-centrifugation supernatant was collected. After the concentration of exosomes was detected using a bicinchoninic acid (BCA) protein quantitation kit (Solarbio, Cat. PC0020), the exosomes were stored in a freezer at -80°C.

The characteristics of exosomes were identified using three approaches: First, the morphology of exosomes was observed by transmission electron microscopy (TEM, G2 spititi FEI, Tecnai, USA). Second, Nanoparticle tracking analysis (NTA) was performed to measure the diameter range of exosomes using ZetaView Particle Metrix (ZetaView PMX 110, Particle Metrix, Germany). Third, the expression of surface markers of exosomes - CD81 (Cat. ab79559), CD63 (Cat. ab193349) and TSG101 (Cat. ab125011) (all from Abcam, United Kingdom) were detected by Western blot analysis.

### Labeling of exosomes and uptake by HUVECs

The exosomes derived from OSCC cells were labeled with PKH 67 fluorescent dye (Umibio, Cat. UR52303) following the manufacturer’s protocol. Briefly, PKH 67 fluorescent dye was prepared according to the instructions and then added to the purified exosomes, after which it was shaken and incubated at room temperature for 10 min. PBS was added to the mixture, which was extracted and purified again in the same way. Subsequently, the precipitate was resuspended in PBS and co-cultured with HUVECs at 37°C for 24 h. Images of ingested exosomes were obtained using an inverted fluorescence microscope (Olympus, Japan).

### Cell Counting Kit-8 assay (CCK8 assay)

A Cell Counting Kit-8 (Dojindo, Cat. CK04, Japan) was carried out to measure cell viability. In all, 5 × 10^3^ HUVECs/well were plated into 96-well plates and incubated with the desired concentrations of phenformin, exosomes (30 μg/mL) or PBS (control). Five wells were designated for each group. At different time points as indicated, 10 μL CCK8 working solution was added to each well, after which the cells were incubated for 2 h at 37°C. The optical absorbance was then measured at a wavelength of 450 nm.

### Transwell migration assays

Transwell migration assays were used to determine the effects of Phen-Exo on the ability to recruit HUVECs. First, 24-well transwell plates containing 8 μm pore size polycarbonate membrane permeable chambers (Corning, Cat. CLS3,422, USA) were selected for the experiment. 200 μL Endothelial Cell Medium containing 1% FBS and exosomes (30 μg/mL) were added to the upper chamber and 500 μL Endothelial Cell Medium containing 5% FBS were placed to the lower chamber. Next, 5 × 10^4^ HUVECs were seeded into the upper chamber. After incubation for 24 h, cells under the polycarbonate membrane were fixed with 4% paraformaldehyde (Solarbio, Cat. P1110) for 10 min and then stained with 0.5% crystal violet (Solarbio, Cat. 8470) for 7 min. After removing the cells at the upper surface of the membrane, the stained cells were recorded and counted at high magnification using a microscope.

### Scratch wound healing assays

Scratch wound healing assays were performed as an alternative approach to detect the migration ability of HUVECs. 3 × 10^4^ HUVECs/well were seeded in six-well plates. When the fusion rate reached around 90%, the growth medium was replaced with serum-free DMEM for 24 h. A scratch mark was generated vertically on the cell surface using a 200 μL sterile pipette tip. After washing three times with PBS, exosomes (30 μg/mL) were added into the medium. The images of scratches were obtained at indicated time points using an optical microscope (Leica, Germany). Analysis of wound closure rates was performed using Image J software (National Institutes of Health, USA).

### Tube formation assays

After Matrigel (Corning, Cat. 354,230) was dissolved at 4°C, 50 μL/well Matrigel was added to 96-well plates on ice and incubated at 37°C for 1 h. 3 ×10^4^ HUVECs were then seeded into each well and incubated at 37°C for 2 h. Depending on the different groups, the medium was changed and appropriate stimuli were given as described in the Figures. The culture plates were incubated at 37°C for 24 h and the tubes formed were captured using a phase contrast microscope (Leica) and were quantified using Image J software. Note that exosome-free serum was used throughout the experiment.

### *In vivo* Matrigel plug angiogenesis assays

Twelve eight-week-old female nude/nude mice (Beijing Vital River Laboratory Animal Technology Co., China) were randomly assigned to four groups. In brief, 2 × 10^6^ HUVECs were suspended in 200 μL Opti-MEM (Gibco, Cat.50985091) with 100 μg exosomes or PBS, and an equal volume of Matrigel was added to the mixture. The above cell mixture was injected subcutaneously on both sides of the back of each nude mouse. The grafts were harvested 2 weeks after the injection and were isolated for photography. Immunohistochemical staining for CD31 (Abcam, Cat. ab28364) and α-SMA (Abcam, Cat. ab108424) and immunofluorescence staining for VEGFA (Abcam, Cat. ab52917) were performed to analyze angiogenesis.

### Chick chorioallantoic membrane (CAM) assays

After 7-day-old fertilized eggs were incubated at 37.8°C for 24 h, a small hole was made in middle of each CAM using tweezers. Next, PBS or VEGFA protein or exosomes (100 μg) were injected into each hole after which the openings were covered with sterile tape. After incubation for 5 d, the CAMs were fixed with 4% paraformaldehyde for 30 min and were excised. The condition of vessel growth in each group was observed using a stereo microscope (Olympus). Three samples were designated in each group.

### Immunohistochemistry (IHC) and immunofluorescence (IF) analysis

The effects of exosomes on angiogenesis-related parameters were assessed by IHC and IF. The tissues obtained were embedded, sectioned and the sections were dewaxed, hydrated, endogenous peroxidase blocked, and subjected to antigen recovery, in that order. Next, each section was incubated with a primary antibody (CD31, α-SMA, VEGFA) overnight at 4°C;. The next day, the sections were incubated with an appropriate secondary antibody, goat anti-rabbit IgG HL (Abcam, Cat. ab6702) or goat anti-mouse IgG HL (Abcam, Cat. ab6708) in the dark at room temperature. After DAB staining and hematoxylin re-staining or labeling of cell nuclei using DAPI (Solarbio, Cat. C0065), analysis of staining was performed using a BX53-DP80 immunofluorescence microscope (Olympus).

### Sequencing analysis of exosomal miRNAs

To explore the expression profiles of miRNAs in exosomes derived from OSCC cells treated with phenformin, microRNA-seq analysis was performed by LC-BioTechnology (Hangzhou, China) with each group having 3 replicates. miRNAs with log2 (Fold change) > 1 and P < 0.05 were considered as differentially expressed miRNAs (DEMs). Volcano plots and Heat maps were generated based on DEMs. The predicted gene targets of top 10 both downregulated and upregulated DEMs in Phen-Exo group were further analyzed using two databases: miRDB and TargetScan, then the KEGG pathway enrichment analysis of predicted targets was carried out to identify top 10 enrichment pathways.

### Western blot analysis

Western blot analysis followed a standard protocol. Briefly, cells were washed three times with ice-cold PBS and were then lysed with radioimmunoprecipitation assay buffer (RIPA buffer, Thermo Fisher Scientific, Cat. 89,900) containing 1% phenylmethylsulfonyl fluoride (PMSF, Thermo Fisher Scientific, Cat. 36,978) and 1% phosphatase inhibitor cocktail (Selleckchem, Cat. B15002, China) for 30 min at 4°C. The mixtures were centrifuged at 20,000 g at 4°C for 10 min and the supernatants were collected. Protein concentrations were measured using a BCA kit. Twenty µg of each extracted cellular protein sample were separated on 12% SDS-polyacrylamide gels and transferred to polyvinylidene fluoride (PVDF) membranes (Thermo Fisher Scientific, Cat. 88,518), according to the manufacturer’s protocol. The membranes were blocked with 5% non-fat milk powder dissolved in Tris-buffered saline containing 0.05% Tween-20 (TBST, Solarbio, Cat. T1081) and then were incubated with the specific primary antibodies (1:1000) noted below overnight at 4°C on a gentle shaker. The next day, following washing three times with TBST buffer for 5 min each, the membranes were incubated with appropriate secondary antibodies (1:5000) for 1 h at room temperature. After washing three times again with TBST buffer for 5 min, bands were detected using an enhanced chemiluminesence detection kit (Solarbio, Cat. SW2050). The relative quantity of proteins was analyzed using Image J software.

The following primary and secondary antibodies were used: CD81 (Abcam, Cat. ab79559, United Kingdom), CD63 (Abcam, Cat. ab193349), TSG101 (Abcam, Cat. ab125011), GAPDH rabbit mAb (Cell Signaling Technology, Cat. #5174, USA), VEGFA (Abcam, Cat. ab52917), goat anti-rabbit IgG HL (Abcam, Cat. ab6702) and goat anti-mouse IgG HL (Abcam, Cat. ab6708).

### Quantitative RT-PCR (qRT-PCR) assays

Total RNAs of HUVECs and exosomes were extracted using Trizol reagent (Thermo Fisher Scientific, Cat. 15596018). The concentration of each RNA was measured using a NanoDrop spectrophotometer (Thermo Fisher Scientific). Each total RNA was reverse transcribed to complementary DNA (cDNA) using a EVO M-MLV kit (Accurate Biology, Cat. AG 11708, China) and All-in-OneTM miRNA qRT-PCR Detection System kit (GeneCopoeia, Cat. HmiRQP0227, China). Next, qRT-PCR assays were performed using a SYBR Green Supermix kit (Biosharp, Cat. 9211, China), an All-in-OneTM miRNA qRT-PCR Detection System Kit and gene-specific primers. PCR reactions were carried out at 95°C for 30 s followed by 40 cycles at 9°C for 5 s and at 60°C for 20 s and ended with an elongation step for 15 s at 72°C. Ct values were used for quantification and relative miRNA expression levels were calculated by the 2^-ΔΔCt^ method normalized by the human housekeeping gene GAPDH for mRNA and RNU6B for miRNA. The primer sequences used are as follows: GAPDH F: 5’-CTCAGTTGCTGAGGAGTCCC-3’, R: 5’-ATCGAGAGAAGGGAGGGCT-3’. RNU6B F: 5’-CTCGCTTCGGCAGCACA-3’, R: 5’-AACGCTTCACGAATTTGCG-3’. VEGFA F: 5′-CCAAAGAAAGATAGAGCAAGACAA-3′, R: 5′-ACACGTCTGCGGATCTTGTA-3′. All primers used for miRNA qRT-PCR were synthesized from GeneCopoeia (Guangzhou, China) and primers used for mRNA qRT-PCR were purchased from Sangon Biotech (Shanghai, China). Three experiments with independent replicates were performed.

### Transfection of HUVECs with specific miRNA mimics/inhibitors and siRNAs of VEGFA

HUVECs at a density of 60% were transfected with specific miRNA mimics or inhibitor or siRNA of VEGFA using Lipofectamine 3000 (Lp 3000, Thermo Fisher Scientific, Cat. L000001). Briefly, 125 μL Opti-MEM containing 12.5 μL of each mimic or inhibitor or VEGFA siRNA was allowed to stand for 10 min while 7.5 μL Lp 3000 was added to another 125 μL aliquot of Opti-MEM. Those two solutions were mixed and allowed to stand for 10 min at room temperature. The final mixture was added to the HUVECs and then incubated for 6 h before changing the medium. After 24 h, the transfected HUVECs were collected to perform tube formation assays, CCK8 assay, qRT-PCR analyses or Western blot analyses. All miRNA mimics, inhibitors and siRNA of VEGFA were synthesized by GenePharma (Suzhou, China). The oligo sequences of 3 independent VEGFA siRNAs: F: 5’-GAUAGAGCAAGACAAGAAATT-3’, R: 5’-UUUCUUGUCUUGCUCUAUCTT-3’; F: 5’-GGCAGCUUGAGUUAAACGATT-3’, R: 5’-UCGUUUAACUCAAGCUGCCTT-3’; F: 5’-CCAUGCAGAUUAUGCGGAUTT-3’, R: 5’-AUCCGCAUAAUCUGCAUGGTT-3’.

### Statistical analysis

All experiments in this study were repeated three times, and results are presented as means ± standard deviation (SD). Statistical analyses were performed using GraphPad Prism 6 (GraphPad Software Inc., LaJolla, CA, USA). Comparisons between two different experimental or treatment groups were performed using the independent samples t test provided that the t test was satisfied, and when more than two groups with two or one set of independent variables were compared separately, one-way or two-way ANOVA was used to correct for pairwise comparisons. P values < 0.05 are considered statistically significant differences.

## Results

### The inhibition of tube formation by vascular endothelial cells caused by conditioned medium derived from OSCC cells treated with phenformin is abolished by an exosome inhibitor

**Figure 1:**
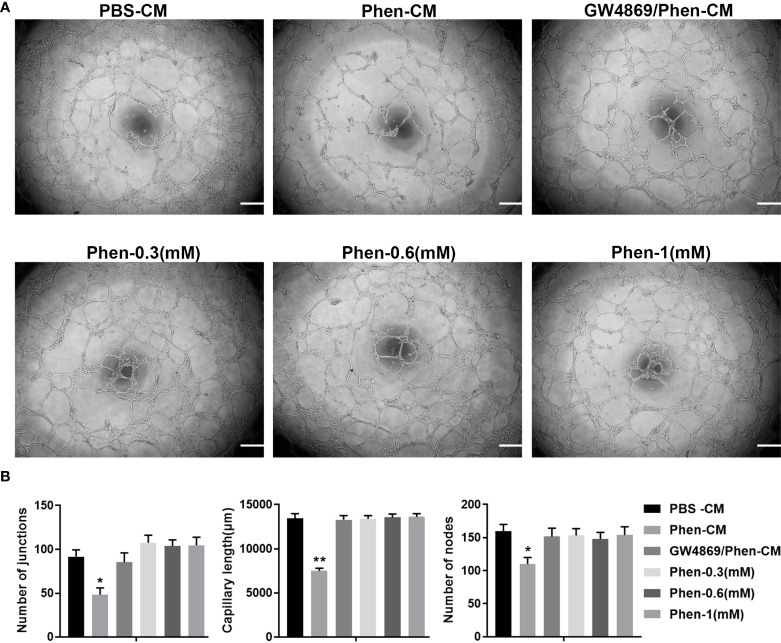
The conditioned medium from CAL 27 OSCC cells treated with phenformin inhibits the tube formation of vascular endothelial cells *in vitro*. **(A)** Matrigel tube formation assays of HUVECs treated with different conditioned medium (CM) collected from CAL 27 cell cultures: the CM from PBS treatment (PBS-CM), the CM from 1 mM phenformin treatment (Phen-CM) and the CM from 20 μM GW4869 plus 1 mM phenformin treatment (GW4869/Phen-CM) or different concentrations of phenformin: 0.3 mM, 0.6 mM and 1 mM. **(B)** Quantification of junctions, capillary length and nodes in the network structures of HUVECs in **(A)**. All experiments were performed three times, and error bars represent means ± SD; P values are indicated with “*”, * indicates P<0.05, ** indicates P<0.01 when comparing Phen-CM group with any other group in panels **(B)** by Student’s t test.

Angiogenesis is essential for cancer progression and metastasis, especially for solid tumors, and anti-angiogenic therapy has been shown to be a promising therapeutic target for anti-cancer treatments (19, 20). Phenformin has recently been shown to suppress cholangiocarcinoma (CCA) cell-mediated angiogenesis (18), and therefore we tested whether phenformin affects OSCC cell-mediated angiogenesis. Conditioned media (CM) collected from CAL 27 OSCC cells treated with PBS or phenformin (Phen) were used for the culture of HUVECs to perform tube formation assays. The results showed that the CM collected from Phenformin (Phen-CM) treated HUVECs suppressed tube formation (**Figure 1A**), decreased the numbers of junctions, length and nodes of blood vessels (**Figure 1B**) compared with the control (PBS-CM). Interestingly, a well-known exosome inhibitor (GW4869), which blocks exosome generation, when added to phenformin to treat CAL 27 cells to obtain CM (GW4869/Phen-CM), counteracted the suppressive effect of Phen-CM on HUVECs. To exclude the direct suppressing role of phenformin on tube formation by HUVECs, three different concentrations of phenformin were added into the medium used to culture HUVECs, and we observed that phenformin didn’t affect the tube formation by HUVECs (**Figures 1A, B**). Taken together, these results suggest that phenformin can stimulate OSCC cells to secrete factors, probably in exosomes, that inhibit tube formation by vascular endothelial cells *in vitro*.

### Characteristics of exosomes extracted from OSCC cells

**Figure 2:**
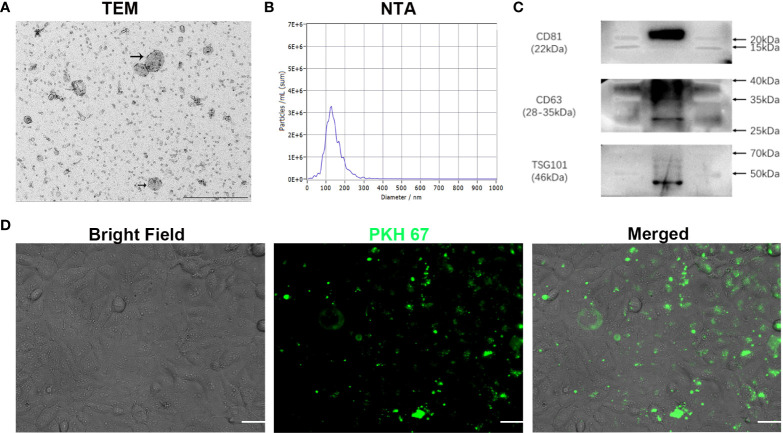
Characterization of exosomes extracted from conditioned medium of OSCC cells *in vitro*. **(A)** The morphology of exosomes was observed using transmission electron microscopy (TEM). Scale bar = 500 nm. **(B)** Particle size distribution of exosomes assessed by nanoparticle tracking analysis (NTA). **(C)** Expression of exosome-specific CD81, CD63 and TSG101 validated by Western blot analysis. **(D)** Efficient uptake of PKH 67-labeled exosomes (green) by HUVECs detected at 24 h. Bright Field shows OSCC cells. Scale bars = 20 μm.

To investigate whether phenformin can inhibit angiogenesis through the regulation of OSCC exosomes, we extracted exosomes from the CM of OSCC cells, and the extracted exosomes were identified by transmission electron microscopy (TEM), nanoparticle tracking analysis (NTA) and Western blot analysis. TEM showed that the morphology of the extracted extracellular vesicles had the typical cup shape of exosomes (**Figure 2A**), and NTA showed that the diameter of those vesicles ranged from 100 nm-150 nm (**Figure 2B**). Western blot analysis showed that those vesicles expressed the surface marker proteins of exosomes: CD81, CD63 and TSG101 (**Figure 2C**). These data conformed that the extracted extracellular vesicles were exosomes. Next, in order to test whether exosomes from OSCC cells have potential effects on vascular endothelial cells, we tested whether those exosomes were taken up by HUVECs. We labeled the exosomes with PKH 67 green fluorescent dye and added them into the medium of HUVECs. After 24 h of incubation, the labeled exosomes (green) were effectively taken up by HUVECs (**Figure 2D**).

### Exosomes derived from phenformin-treated OSCC cells inhibit the proliferation, migration and tube formation of vascular endothelial cells *in vitro*

**Figure 3:**
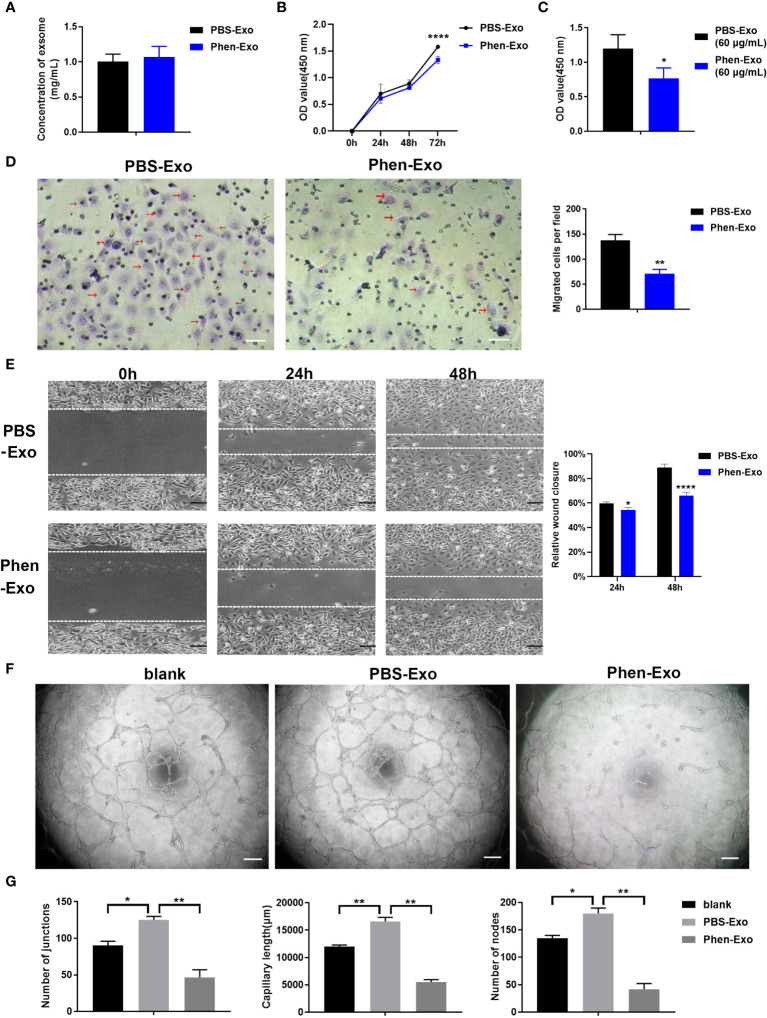
Exosomes derived from OSCC cells treated with phenformin (Phen-Exo) inhibit the proliferation, migration and tube formation of vascular endothelial cells *in vitro*. **(A)** The concentration of exosomes extracted from the conditioned medium of CAL 27 cells treated with PBS (PBS-Exo) or Phenformin (Phen-Exo) measured using a BCA protein quantitation kit. **(B)** HUVECs were treated with PBS-Exo (30 μg/mL) or Phen-Exo (30 μg/mL), then were collected at the different time points indicated and analyzed by the CCK8 assay for cell viability. **(C)** HUVECs were treated with PBS-Exo (60 μg/mL) or Phen-Exo (60 μg/mL), then were collected at 72 h and analyzed by the CCK8 assay for cell viability. **(D)** (Left) images of migrated HUVECs (red arrows) through membranes at 24 h after treatment with PBS-Exo (30 μg/mL) or Phen-Exo (30 μg/mL). Scale bars = 50 μm, and (Right) quantification of the number of migrated cells per field. **(E)** (Left) images of HUVECs cultured with PBS-Exo (30 μg/mL) or Phen-Exo (30 μg/mL) at 0 h, 24 h and 48 h after scratching. Scale bars = 200 μm, and (Right) bar graph of quantification of the rate of scratch closure (%). **(F)** Matrigel tube formation assay of HUVECs treated with PBS (blank), PBS-Exo (30 μg/mL) or Phen-Exo (30 μg/mL). Scale bars = 200 μm. **(G)** Quantification of junctions, capillary length and nodes in the network structures of HUVECs in **(F)**. All experiments were performed three times, and error bars represent means ± SD in each group; P values are indicated with “*”, * indicates P<0.05, ** indicates P<0.01, **** indicates P<0.0001.

The above results showed that the CM derived from phenformin-treated OSCC cells inhibit endothelial cell tube formation likely through its component exosomes. Therefore, we first examined whether phenformin could affect the secretion level of exosomes into CM by OSCC cells, since we observed that OSCC cell growth were clearly inhibited by phenformin (**Supplementary Figure 1**). The total protein concentrations of exosomes, which has been usually used for quantifying exosomes (21, 22), extracted from CM derived from phenformin-treated OSCC cells (Phen-Exo) or from control cells with PBS treatment (PBS-Exo) were measured, and the results showed no significant difference between the two groups (**Figure 3A**), which suggests that phenformin treatment didn’t change the secretion level of exosomes from OSCC cells. We then investigated the effect of exosomes derived from phenformin-treated OSCC cells (Phen-Exo) on angiogenesis by adding same protein concentration of exosomes derived from control group (PBS-Exo). Since angiogenesis is closely related to the proliferation of vascular endothelial cells, we first examined whether Phen-Exo affect the growth of endothelial cells using CCK8 assays to evaluate cell viability after incubation with Phen-Exo or with PBS-Exo (30 μg/mL). We found that Phen-Exo inhibited the growth of endothelial cells *in vitro* compared with the control (**Figures 3B, C**).

Next, trans-well migration assays were carried out to investigate the effects of Phen-Exo on the migratory capacity of vascular endothelial cells, which is another important factor involved in angiogenesis. We found that the number of cells migrating to the bottom compartment in the Phen-Exo group was significantly less than that of the control PBS-Exo group (**Figure 3D**). As an alternative approach, scratch wound healing assays were performed to validate the suppressing effect of Phen-Exo on the migratory ability of HUVECs. The *in vitro* wound healing results showed that HUVECs treated with 30 μg/mL Phen-Exo had a lower rate of wound closure than the PBS-Exo treated group (**Figure 3E**). These results suggest that exosomes derived from phenformin-treated OSCC cells can inhibit the migration of vascular endothelial cells *in vitro*.

Finally, we tested whether Phen-Exo could directly affect the tube formation of vascular endothelial cells *in vitro*. As shown in **Figures 3F, G**, the PBS-Exo treated group formed more junctions, longer network structures and more nodes than the blank group, however, all these parameters of tube formation were reduced in the Phen-Exo treated group compared to the PBS-Exo group. These results demonstrated that exosomes secreted from OSCC cells treated with phenformin can inhibit vascular endothelial cell growth, migration and angiogenesis *in vitro*.

### Exosomes derived from phenformin-treated OSCC cells inhibit angiogenesis of vascular endothelial cells *in vivo*

**Figure 4:**
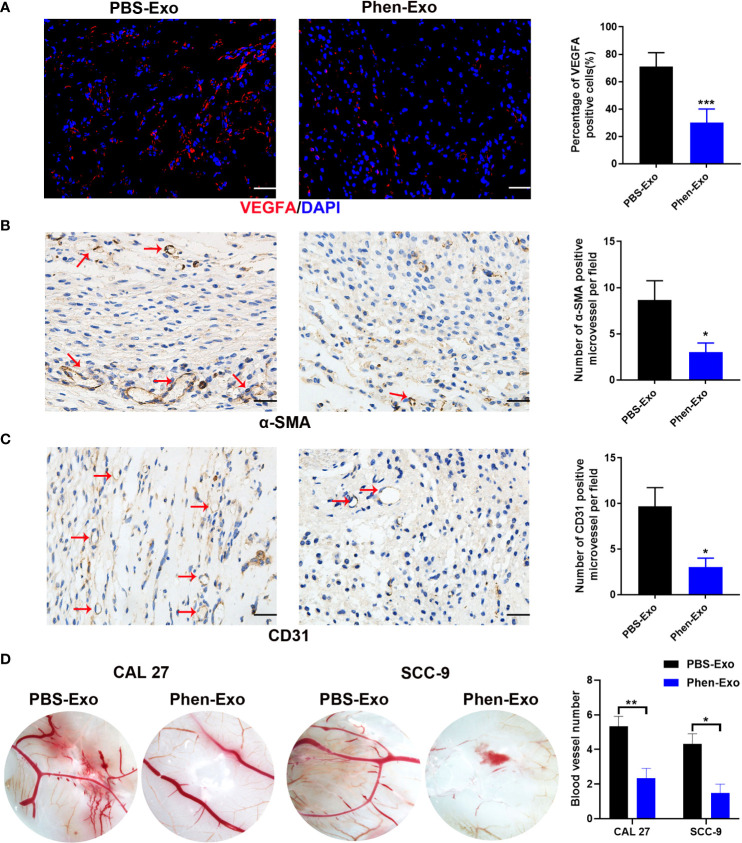
Exosomes derived from OSCC cells treated with phenformin (Phen-Exo) inhibit angiogenesis *in vivo*. **(A–C)** Immunofluorescence staining for VEGFA and IHC staining for α-SMA and CD31 in grafts formed following the injection of HUVECs together with PBS-Exo or Phen-Exo derived from CAL 27 OSCC cells. Blood vessels with α-SMA and CD31 positive cells in the grafts are indicated by red arrows. Bar graphs on the right show quantification of the numbers of VEGFA, α-SMA and CD31 positive cells. Scale bars = 50 μm in A, scale bars = 20 μm in **(B–D)** Images of blood vessels formed in CAM assays with treatment of PBS-Exo (100 μg/injection site) or Phen-Exo (100 μg/injection site) derived from CAL 27 cells or SCC-9 cells. Bar graph on the right shows quantification of blood vessel numbers observed in CAM assays. All experiments were performed three times, and error bars represent means ± SD in each group; P values are indicated with “*”, * indicates P<0.05, ** indicates P<0.01, *** indicates P<0.001.

To validate the above *in vitro* findings *in vivo*, two approaches were carried out as follows: First, we performed matrigel plug angiogenesis assays, a classic murine model to examine the angiogenic ability of endothelial cells (23). A mixture of exosomes and HUVECs was injected subcutaneously into the dorsal skin of nude mice. After 2 weeks, the grafts were collected for evaluation of blood vessel formation by staining the endothelial cell markers VEGFA, α-SMA and CD31 (**Figures 4A–C**). We observed that there were fewer VEGFA, α-SMA and CD31 positive cells in the grafts of the Phen-Exo treated group (**Figures 4A–C**), and especially, a lower number of blood vessel structures, indicated by α-SMA or CD31 staining (red arrows, **Figures 4B, C**), were seen in grafts of the Phen-Exo treated group. This result was further validated by grafting of Phen-Exo derived from another OSCC cell line, SCC-9 (**Supplementary Figures 2A-C**).

Second, CAM assays, a classic assay for studying angiogenesis *in vivo* (24), were performed to evaluate the effects of exosomes derived from phenformin-treated OSCC cells on angiogenesis. Fertilized chicken eggs were incubated at 37.8°C; for 8 days, after which Phen-Exo or PBS (control) were injected *via* small holes in the middle of each CAM. After further incubation for 48 h, the CAMs were excised and the number of micro-vessels around each injection area were calculated. The results showed that the number of micro-vessels formed was decreased in the Phen-Exo-treated group (**Figure 4D**). This result was further validated by the injection of Phen-Exo from another OSCC cell line, SCC-9 (**Figure 4D**).

In summary, the above results demonstrated that exosomes derived from phenformin-treated OSCC cells can inhibit angiogenesis *in vivo*.

### Exosomes derived from phenformin-treated OSCC cells express high levels of miR-1246 and miR-205

**Figure 5:**
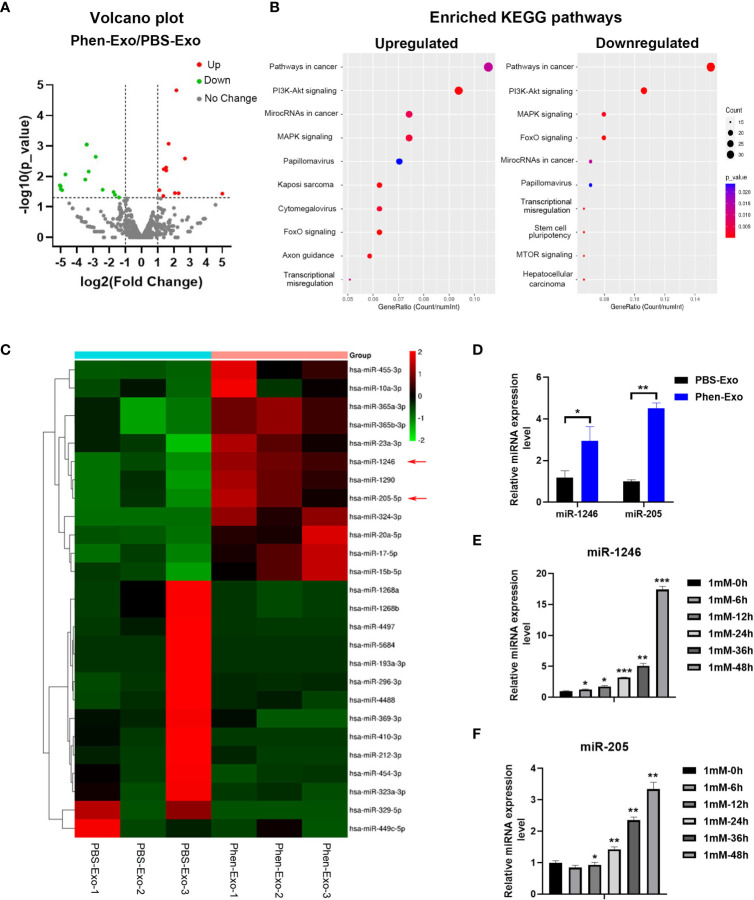
Phen-Exo derived from OSCC cells express high levels of miR-205 and miR-1246. **(A)** Volcano plot presenting differentially expressed miRNAs with a ≥2 fold difference between Phen-Exo and PBS-Exo. **(B)** The enriched KEGG pathway analysis of predicted target genes of top 10 up-regulated (Left) or down-regulated (Right) differentially expressed miRNAs. The X axis shows the enrichment factor; the left Y axis shows the top 10 positive KEGG pathway names (detail description of pathway name showed in **Supplementary Table 1**). The darker the color represents the smaller the p value. Bubble size indicates the number of differentially expressed miRNAs. **(C)** Heat map showing profiles of significantly upregulated and significantly down-regulated miRNAs in Phen-Exo vesus PBS-Exo. Red arrows indicate miR-1246 and miR-205. **(D)** qRT-PCR analysis of PBS-Exo and Phen-Exo derived from CAL 27 OSCC cells for expression levels of miR-1246 and miR-205. **(E, F)** qRT-PCR analysis of CAL 27 cells treated with 1 mM phenformin at 0, 6, 12, 24, 36, 48 h for expression levels of miR-1246 **(E)** and miR-205 **(F)**. **(D–F)** Relative expression levels were normalized by the human RNU6B gene. The error bars represent means ± SD in each group; P values are indicated with “*”, * indicates P<0.05, ** indicates P<0.01, *** indicates P<0.001.

Next, we characterized the underlying mechanism whereby exosomes derived from phenformin-treated OSCC cells inhibit angiogenesis. An increasing number of studies has shown that miRNAs play a key role in exosome functions as essential mediators for intercellular communications (25), and therefore miRNA sequencing was performed to evaluate the expression profiles of miRNAs encapsulated in Phen-Exo vesus control PBS-Exo derived from OSCC cells. A Volcano plot was generated to identify differentially expressed miRNAs (DEMs) with statistical significance (P<0.05) (**Figure 5A**), A Venn plot showed that there were 12 up-regulated and 15 down-regulated DEMs in the Phen-Exo group compared with the PBS-Exo group (**Supplementary Figure 3**). The enriched KEGG pathways of predicted gene targets of top 10 up-regulated and down-regulated DEMs were analyzed and top 10 enriched pathways of from either up-regulated or down-regulated DEMS were shown in **Figure 5B** (detail description of pathways shown in **Supplementary Table 1**). It was not surprised that “Pathways in cancer” ranked No.1 pathway. And the analysis also showed that these enriched pathways, such as PI3K/Akt pathway, MAPK pathway, mTOR pathway and TGF-beta pathway, targeted by these DEMs, are involved in cell proliferation, migration, as well as angiogenesis (**Figure 5B**). The heat map analysis further revealed that the name of up-regulated and down-regulated DEMs (**Figure 5C**). Since we observed a high variation in the down-regulated miRNAs among the 3 replicate Phen-Exo samples (**Figure 5C**), we decided to investigate the 12 up-regulated miRNAs. We reviewed the literature to study which miRNA(s) among those 12 miRNAs is related to angiogenesis and found that miR-1246 has recently been shown to be an exosome component that suppresses the angiogenic function of endothelial cells *in vitro* and *in vivo* (26). Further, miR-205 has also been reported to be involved in angiogenesis (27, 28), and therefore both miR-1246 and miR-205 (red arrows in **Figure 5C**) were selected for further validation.

First, qRT-PCR analysis verified the higher expression levels of both miR-1246 and miR-205 in Phen-Exo (**Figure 5D**) derived from OSCC cells compared to the control PBS-Exo group. We then examined whether the increased levels of miR-1246 and/or miR-205 in Phen-Exo is because phenformin directly enhances OSCC cells to express miR-1246 and/or miR-205. CAL 27 OSCC cells were treated with phenformin and were collected at different time points for analysis of miR-1246 and miR-205 expression. We found that phenformin treatment promoted the expression of both miR-1246 and miR-205 in CAL 27 cells (**Figures 5E, F**). In sum, these results suggest that phenformin induces miR-1246 and miR-205 expression in OSCC cells, which likely results in the enrichment of those two miRNAs in exosomes secreted from phenformin-treated OSCC cells.

### Both miR-1246 and miR-205 target VEGFA to inhibit angiogenesis

**Figure 6:**
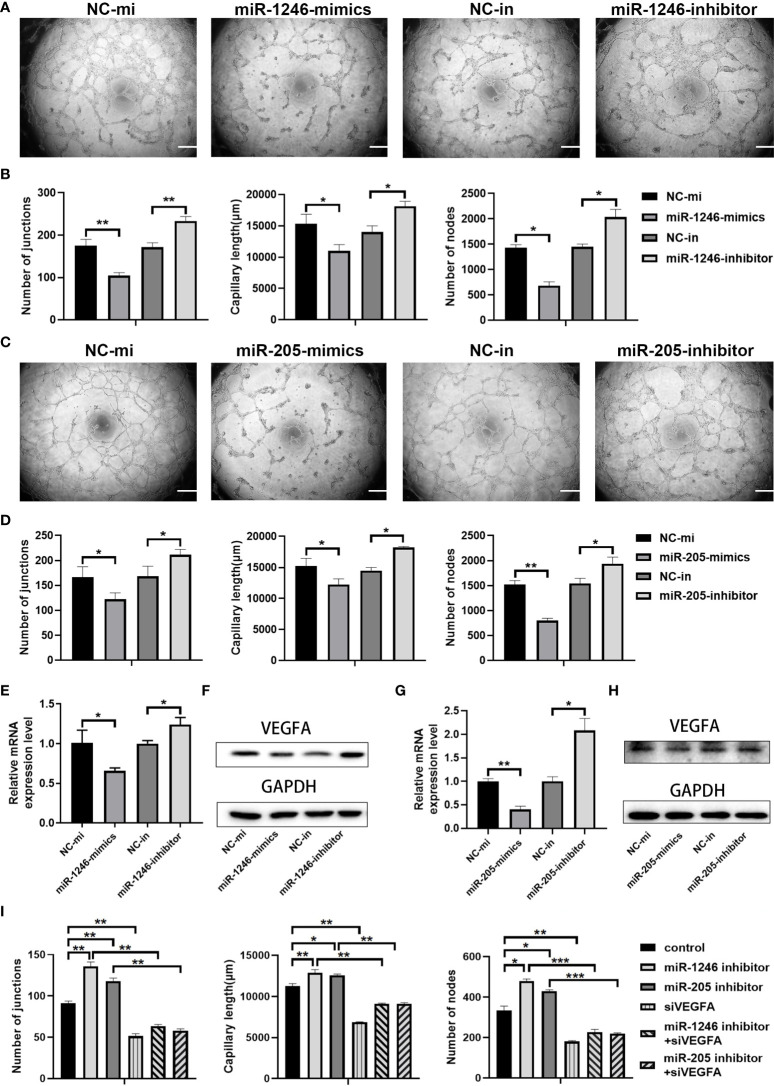
miR-1246 and miR-205 inhibit tube formation by vascular endothelial cells *via* the suppression of VEGFA expression. **(A)** Images of tube formation by HUVECs at 24 h after transfection with miR-1246 mimics or miR-1246 inhibitors or corresponding controls (NC-mi or NC-in). **(B)** Quantification of the numbers of junctions, capillary tube length and nodes in the network structures of HUVECs in **(A. C)** Images of tube formation of HUVECs at 24 h after transfection with miR-205 mimics or miR-205 inhibitors or corresponding controls (NC-mi or NC-in). **(D)** Quantification of the numbers of junctions, capillary tube length and nodes in the network structures of HUVECs in **(C)**. **(E–H)** Expression of VEGFA analyzed by qRT-PCR **(E, G)** and Western blot **(F, H)** in HUVECs at 24 h after transfection with miR-1246 mimics or inhibitors **(E, F)** or miR-205 mimics or inhibitors **(G, H)** or corresponding controls (NC-mi or NC-in) and quantification of the expression levels of VEGFA protein shown in **Supplementary Figure 6**. **(I)** Quantification of the number of junctions, capillary tube length and nodes in the network structures of tube formation (shown in Supplementary Figure 7) by HUVECs at 24 h after transfection with the following conditions as indicated: control vehicles, miR-1246 inhibitor, miR-205 inhibitor, siVEGFA, miR-1246 inhibitor plus siVEGFA, and miR-205 inhibitor plus siVEGFA, All experiments were performed three times, and error bars represent means ± SD; P values are indicated with “*”, * indicates P<0.05, ** indicates P<0.01, *** indicates P<0.001.

We then evaluated whether miR-1246 and/or miR-205 directly affect endothelial cell angiogenesis *in vitro.* HUVECs were transfected with miRNA mimics to increase their expression or with miRNA inhibitors to suppress their expression. Twenty-four h after transfection, qRT-PCR analysis revealed that the expression of miR-1246 and miR-205 was efficiently elevated by transfection of their specific mimics or was significantly decreased by transfection of their specific inhibitors in HUVECs compared to the corresponding control (**Supplementary Figures 4A, B**). First, we examined that the effect of miR-1246 or miR-205 on the growth of HUVECs, and we found that both miR-1246 and miR-205 negatively regulate endothelial cell growth (**Supplementary Figure 5**). Then, *in vitro* tube formation assays were then carried out with the transfected HUVECs to investigate the effects of the target miRNAs on angiogenesis. The results showed that tube formation was inhibited in HUVECs transfected with miR-1246 or miR-205 mimics, but tube formation was increased in HUVECS transfected with the inhibitors (**Figures 6A–D**). Taking these data together, both miR-1246 and miR-205 can suppress the tube formation of HUVECs.

Next, we investigated how those two miRNAs regulate angiogenesis. Previous reports showed that miR-1246 and miR-205 can regulate angiogenesis through VEGFA (26, 28, 29), which is the most well-known member of the VEGF family and is a crucial modulator of vascular permeability and angiogenesis (30, 31). Changes of VEGFA expression in the transfected HUVECs were detected both by qRT-PCR analysis (**Figures 6E, G**) and by Western blot analysis (**Figures 6F, H**, **Supplementary Figure 6**). As shown in **Figures 6E–H** and **Supplementary Figure 6**, VEGFA mRNA and protein expression levels were decreased both in the miR-1246-mimics group and in the miR-205-mimics group, while opposite results were seen in the inhibitor groups. Furthermore, either miR-1246 or miR-205 inhibitors were transfected into HUVECs with knockdown of VEGFA by siRNA (siVEGFA), and we found that the increased tube formation of HUVECs induced by either miR-1246 or miR-205 inhibitors was abolished by knockdown of VEGFA (**Supplementary Figure 7** and **Figure 6I**). Taken these data together, it indicated that both miR-1246 and miR-205 negatively control vascular endothelial cell angiogenesis by regulation of VEGFA expression.

## Discussion

Metformin, the most commonly prescribed drug for type II diabetes, has been known to have potential antitumor activities in a large variety of tumors (32). Phenformin, another biguanide drug used to treat type II diabetes mellitus, was removed from clinical use due to a high risk of fatal lactic acidosis (33). However, an increasing number of studies has shown that phenformin has a higher antitumor effectiveness due to its greater absorption by tumor cells and its higher potency and tissue bioavailability, compared to metformin (34). Here, we show a mechanism through which phenformin treated OSCC cells, which secrete exosomes with specific miRNAs, inhibit the tube formation of vascular endothelial cells, which may serve as a new strategy for oral cancer therapy. Indeed, previous studies have focused on the direct role of phenformin as well as metformin in cancer cells, but there have been few studies on their effects in the tumor microenvironment. Although Kim et al. reported the antitumor activities of phenformin to inhibit myeloid-derived suppressor cells in various melanoma models, those effects were independent of exosome secretion (35). Metformin has been shown to play a role in facilitating the release of exosomes and in optimizing the therapeutic potential of exosomes in intervertebral disc degeneration (14). Our current study adds another mechanism by which phenformin plays an antitumor function through the induced secretion of exosomes by cancer cells. Exosomes are extracellular nanovesicles that deliver diverse cargoes to cells and participate in cell communications. Exosome therapies have a number of potential advantages, such as lower manufacturing costs, higher stability and more convenient sterilization, storage and infusion treatment (36). We demonstrate that exosomes secreted from OSCC cells treated with phenformin inhibit angiogenesis. Exosomes encapsulated with diverse bioactive cargoes, including miRNAs, are transferred to endothelial cells to regulate cell function and angiogenesis processes (37, 38).

To understand how exosomes derived from OSCC cells treated with phenformin inhibit angiogenesis, we performed exosomal miRNA sequencing and identified that both miR-1246 and miR-205 are abundant in Phen-Exo from OSCC cells, verified by qRT-PCR analysis. Our further study found that phenformin treatment could enhance the expression levels of miR-1246 and miR-205 in OSCC cells, which mainly resulted in the high expression of miR-1246 and miR-205 in Phen-Exo derived from OSCC cells. Exosomes originate from multivesicular bodies (MVBs), a cellular late endosome that contains intraluminal vesicles (ILVs) within the endosomal compartment (39, 40). A previous study demonstrated that metformin increased the release of exosomes by human glioblastoma cells (15). Metformin promotes the fusion of MVBs with the plasma membrane *via* SNAP29 phosphorylation. Further, metformin facilitates the co-localization of ITIH4 and SNPA29, thereby facilitating the cargo transport in exosomes and metformin-induced autophagy is associated with the production and release of exosomes (14). Phenformin has been shown to induce cell autophagy in cholangiocarcinoma cells (41). However, whether phenformin-induced autophagy is associated with the production and release of exosomes needs to be further studied. Metformin has been reported to exert its anticancer effects *via* miRNAs due to the induction of DICER expression (42). A previous study demonstrated that phenformin increases the expression of miR-124, 137 and let-7 although the underlying mechanism was not investigated (43). Therefore, it will be interesting to determine how phenformin controls miR-1246 and miR-205 expression in OSCC cells in the future.

The miR-205 has been reported to directly bind the 3’ UTR region of VEGFA mRNA to negatively control its expression in several cell types, such as breast cancer, colorectal cancer and bladder cancer as well as endothelial cells (44–47). For miR-1246, our previous study together other publications have already showed that miR-1246 didn’t directly bind VEGFA mRNA, but can indirectly target VEGF through miR-1246/ACE signal pathways to inhibit VEGF expression resulting in suppressing angiogenesis of endothelial cells (26, 29, 48). In the present study, we showed both VEGFA mRNA and protein expression levels were significantly decreased in both the miR-1246-mimics and the miR-205-mimics group, while opposite results were seen in the corresponding inhibitor groups. Moreover, we showed that increased tube formation induced by both miR-1246 and miR-205 inhibitors were abolished by knockdown of VEGFA in endothelial cells. Taken this together, we could conclude that both miR-1246 and miR-205 suppress endothelial cell angiogenesis by decreasing the expression of VEGFA.

In conclusion, this study revealed that phenformin can inhibit angiogenesis by regulating the levels of miR-1246 and miR-205 in extracellular exosomes secreted by OSCC cells, which suggests that phenformin has the potential to alter the tumor microenvironment to antagonize the growth of OSCCs, which may provide a theoretical basis for the development of new strategies to treat OSCCs in the future.

## Data availability statement

The original contributions presented in the study are included in the article/**Supplementary Material**. Further inquiries can be directed to the corresponding authors.

## Ethics statement

The animal study was reviewed and approved by Ethics Committee of the Hospital of Stomatology, Shandong University.

## Author contributions

JM and XW conceived and designed the study, and provided acquisition, analysis and interpretation of data. DZ, SW, GL, PL, HD, JS, CL, XL, QZ, and FB collected the data and conducted the statistical analyses. JM and XW performed the development of methodology, writing and review of the manuscript. All authors read and approved the final version of the manuscript.

## Funding

This work was supported by the General Program of National Natural Science Foundation of China (82073470) and the Key Research and Development Program of Shandong Province (ZR2019ZD36) to XW.

## Conflict of interest

The authors declare that the research was conducted in the absence of any commercial or financial relationships that could be construed as a potential conflict of interest.

## Publisher’s note

All claims expressed in this article are solely those of the authors and do not necessarily represent those of their affiliated organizations, or those of the publisher, the editors and the reviewers. Any product that may be evaluated in this article, or claim that may be made by its manufacturer, is not guaranteed or endorsed by the publisher.
