# Supplementary material for: Phenformin suppresses angiogenesis through the regulation of exosomal microRNA-1246 and microRNA-205 levels derived from oral squamous cell carcinoma cells
Source: Front Oncol. 2022 Sep 8;12:943477. doi: 10.3389/fonc.2022.943477 (PMC9492847; doi:10.3389/fonc.2022.943477)
Supplement: Supplementary file 1 [file DataSheet_1.docx]

Supplementary Material

## **Supplementary Figures**

**
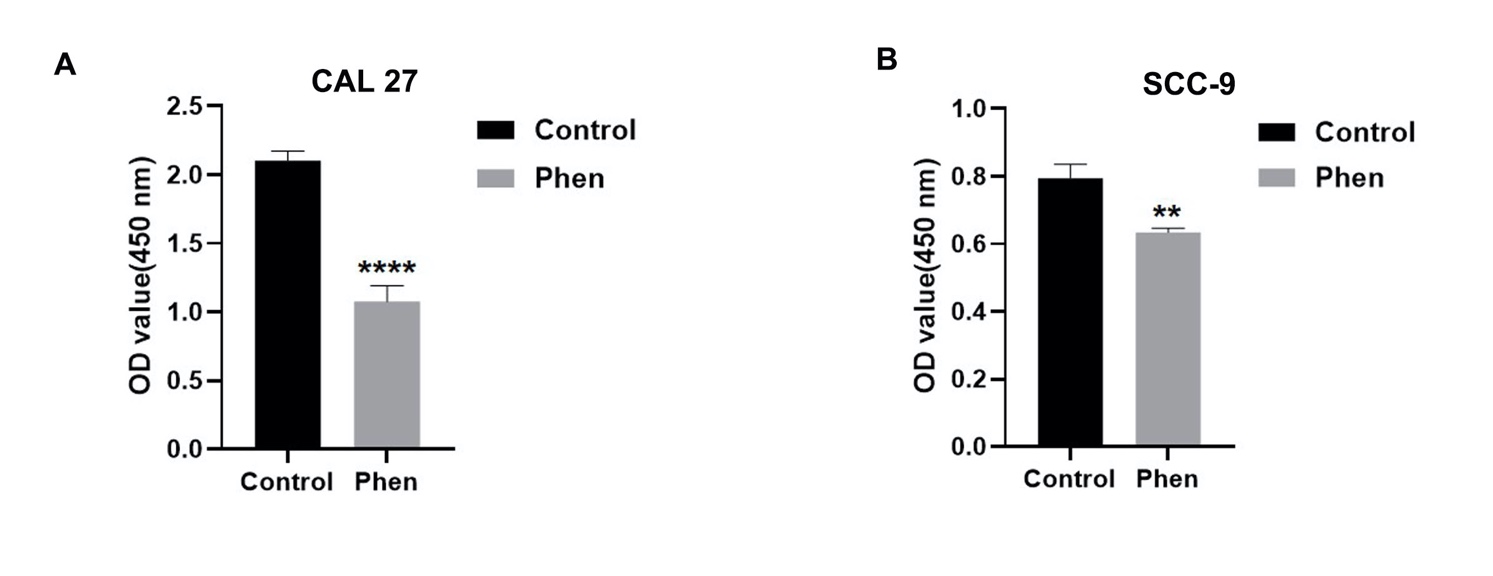
**

**Supplementary Figure 1. Phenformin suppresses OSCC cell growth *in vitro*.**

**A-B** Both CAL 27(**A**) and SCC-9 (**B**) OSCC cells were treated either 1mM phenformin (Phen) or PBS as control for 24 hours, then cells were collected for analysis of cell viability with CCK8 assay kit. All experiments were performed three times, and error bars represent means ± SD in each group; P values are indicated with “*”, ** :p<0.01; ****:p<0.0001

**
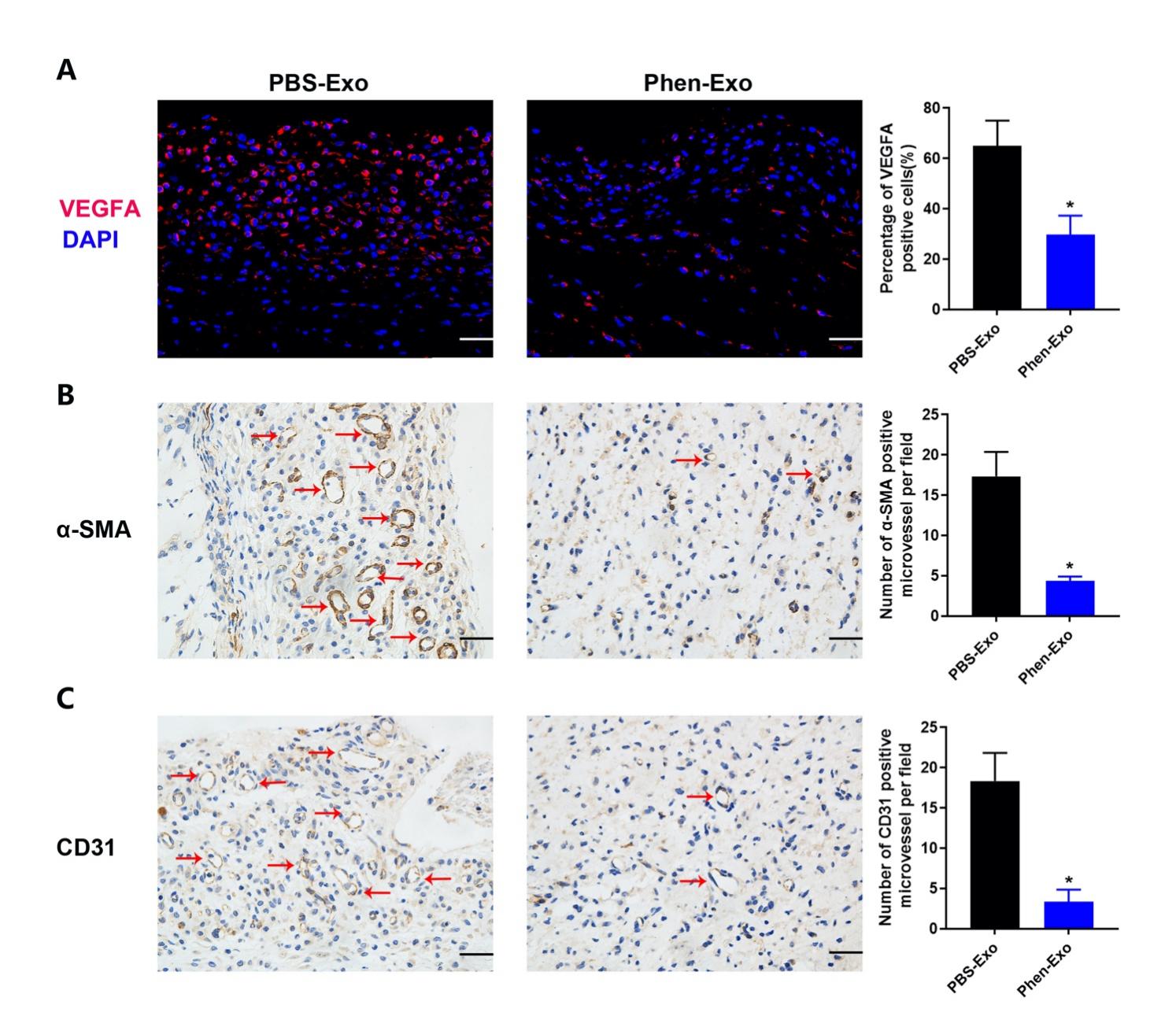
**

**Supplementary Figure 2. Exosomes derived from phenformin-treated OSCC cells inhibit angiogenesis of vascular endothelial cells *in vivo*.**

**A-C** Immunofluorescence staining for VEGFA and immunohistochemical staining for α-SMA and CD31 in grafts formed after the injection of HUVECs together with PBS-Exo or Phen-Exo derived from SCC-9 cells. Blood vessels with α-SMA and CD31 positive cells in the grafts are indicated by red arrows. Bar graphs on the right show quantification of the numbers of VEGFA, α-SMA, CD31 positive cells. Scale bars = 50 μm in **A**, Scale bars = 20 μm in **B** and **C**. All experiments were performed three times, and error bars represent means ± SD in each group; P values are indicated with “*”, * indicates P<0.05.

**
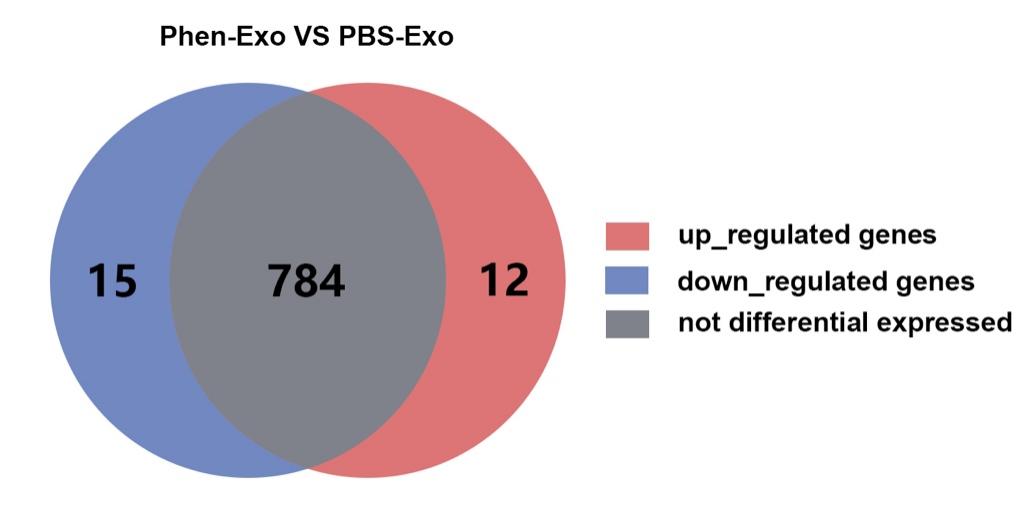
**

**Supplementary Figure 3.** **The Venn plot presents the number of up-regulated, down-regulated and not changed microRNAs in exosomes derived from phenformin treated OSSC cells (Phen-Exo) comparing to the control group (PBS-Exo)**

**
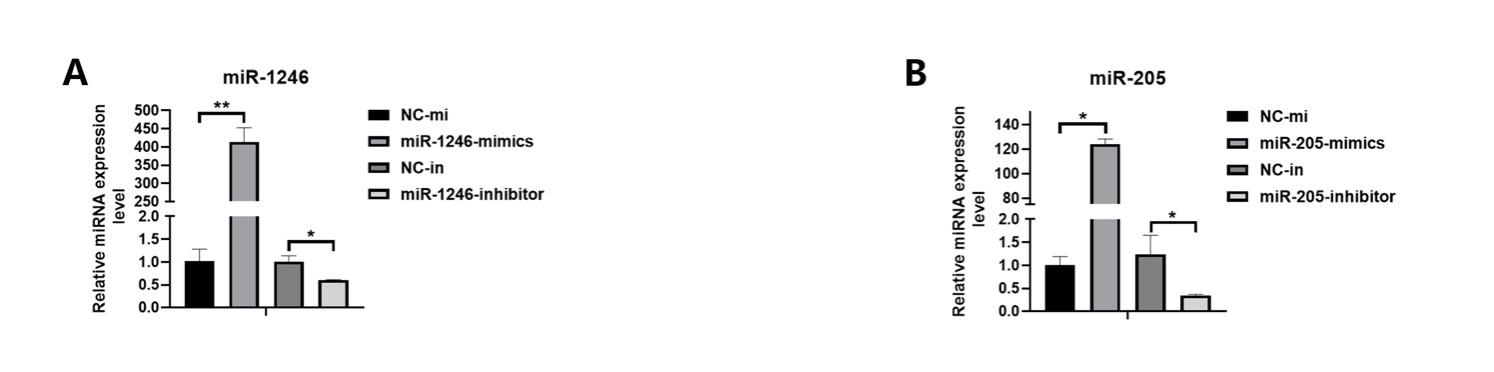
**

**Supplementary Figure 4.** **Transfection of miRNA mimics or inhibitors significantly change the expression levels of corresponding miRNAs in HUVECs.**

**A, B** Expression of miR-1246 (**A**) and miR-205 (**B**) analyzed by qRT-PCR in HUVECs at 24 h after transfection with miR-1246 mimics or inhibitors or miR-205 mimics or inhibitors or corresponding controls (NC-mi or NC-in). Expression levels of miR-1246 or miR-205 were calculated relative to RNU6B expression in each group. All experiments were performed three times, and error bars represent means ± SD in each group; P values are indicated with “*”, * indicates P<0.05, ** indicates

P<0.01.


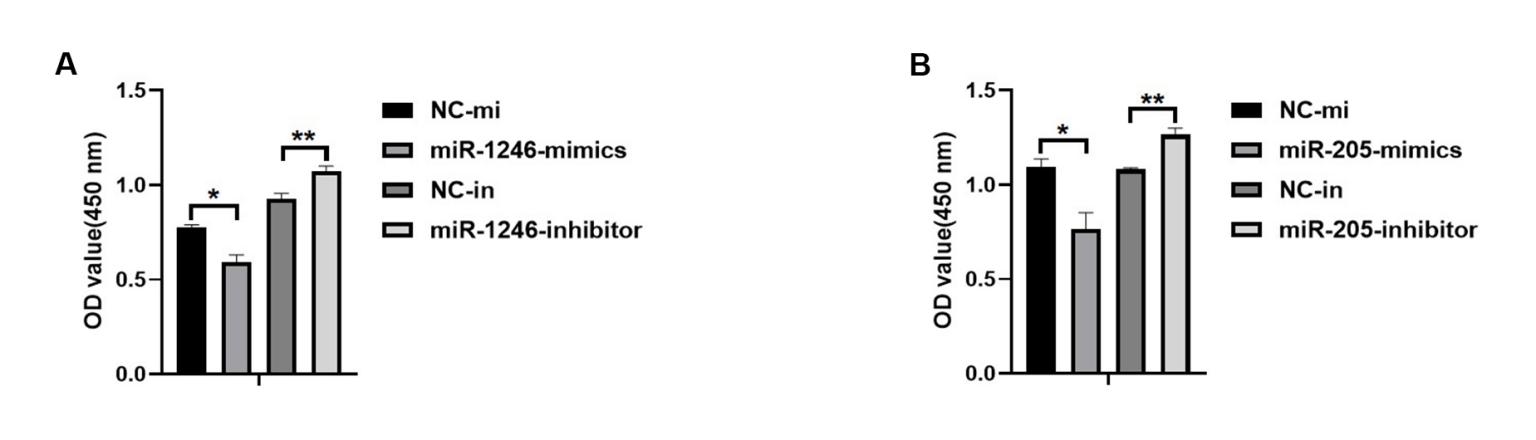


**Supplementary Figure 5. miR-1246 and miR-205 negatively regulate the growth of HUVECs .**

**A, B** HUVECs were transfected with miR-1246 mimics or inhibitors (**A**) or miR-205 mimics or inhibitors (**B**) or corresponding controls (NC-mi or NC-in), then were collected at 48 h and analyzed by the CCK8 assay for cell viability. All experiments were performed three times, and error bars represent means ± SD in each group; P values are indicated with “*”, * indicates P<0.05, ** indicates

P<0.01.


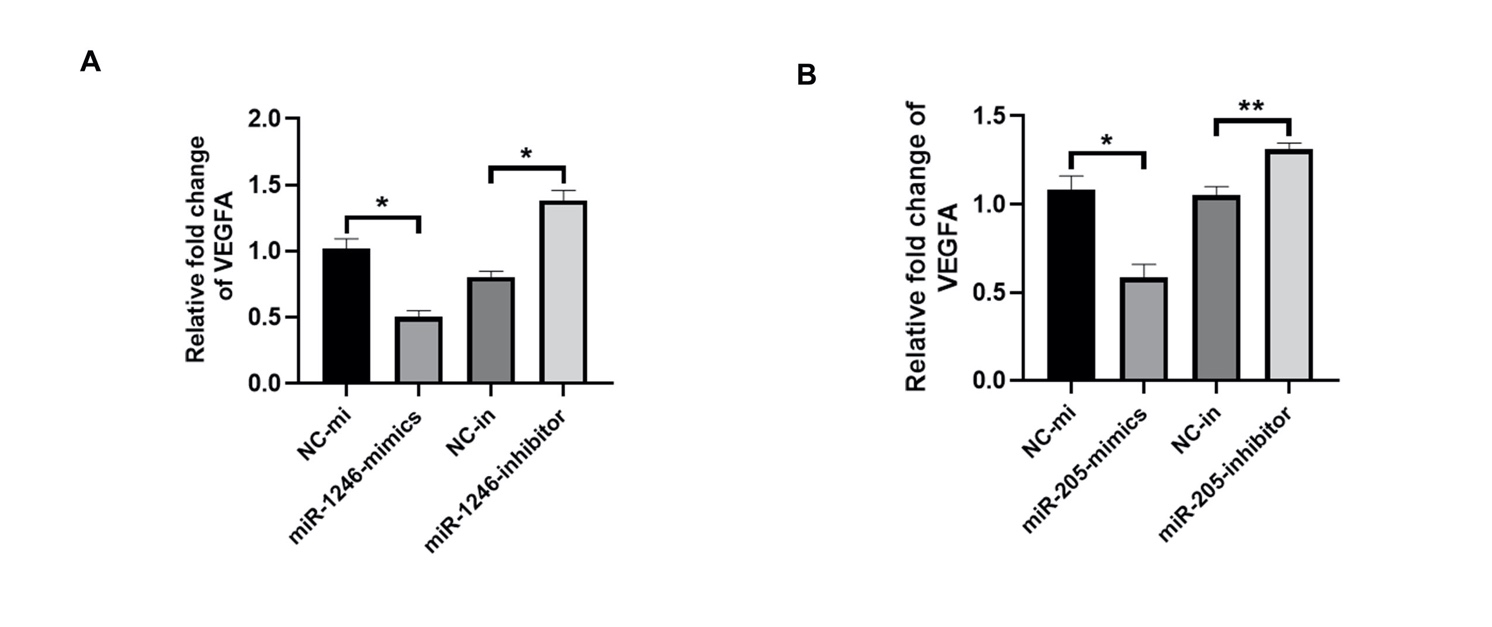


**Supplementary Figure 6. miR-1246 and miR-205 negatively regulate VEGFA protein expression**

Quantification of Western blot analysis in Fig. 6F,H for VEGFA expression in HUVECs at 24 h after transfection with miR-1246 mimics or inhibitors (A) or miR-205 mimics or inhibitors (B) or corresponding controls (NC-mi or NC-in). Relative expression levels of VEGFA were normalized by the human GAPDH gene. All experiments were performed three times, and error bars represent means ± SD; P values are indicated with “*”, * indicates P<0.05, ** indicates P<0.01.


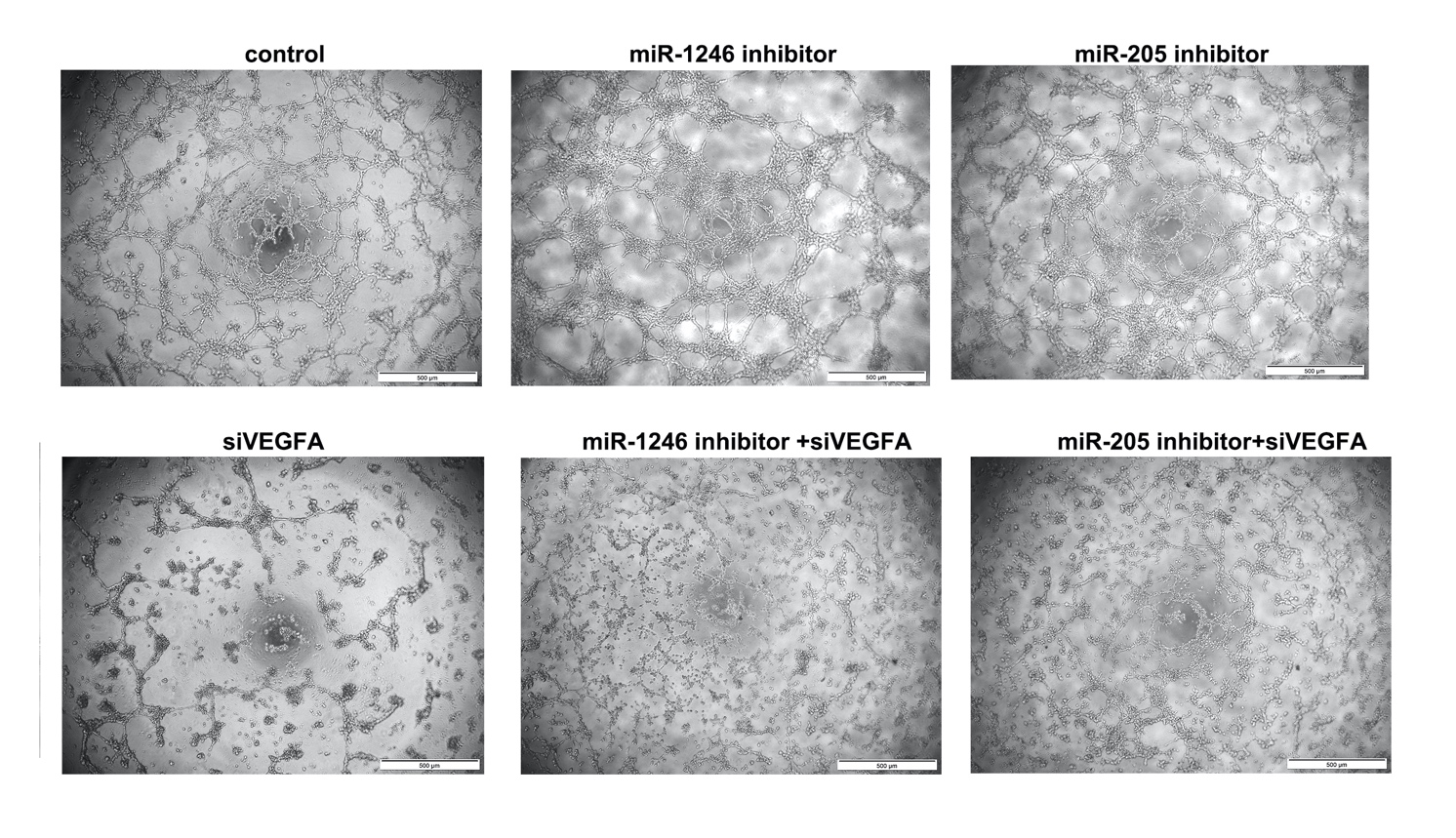


**Supplementary Figure 7. Knockdown of VEGFA counteracted the tube formation induced by miR-1246 and miR-205 inhibitors**

The tube formation of HUVECs at 24 h after transfection with the following conditions as indicated: control vehicles, miR-1246 inhibitor, miR-205 inhibitor, siVEGFA, miR-1246 inhibitor plus siVEGFA, and miR-205 inhibitor plus siVEGFA. Bars=500μm. Quantification of the number of junctions, capillary tube length and nodes in the network structures of tube formation shown in Fig.6I.

**Supplementary Table**

**Supplementary Table 1 Description of pathways showed in Figure 5B**

| **Entry** | **Description of pathways** |
| --- | --- |
| hsa05200 | Pathways in cancer - Homo sapiens (human) |
| hsa04151 | PI3K-Akt signaling pathway - Homo sapiens (human) |
| hsa05206 | MicroRNAs in cancer - Homo sapiens (human) |
| hsa04010 | MAPK signaling pathway - Homo sapiens (human) |
| hsa05165 | Human papillomavirus infection - Homo sapiens (human) |
| hsa05167 | Kaposi sarcoma-associated herpesvirus infection - Homo sapiens (human) |
| hsa05163 | Human cytomegalovirus infection - Homo sapiens (human) |
| hsa04068 | FoxO signaling pathway - Homo sapiens (human) |
| hsa04360 | Axon guidance - Homo sapiens (human) |
| hsa05202 | Transcriptional misregulation in cancer - Homo sapiens (human) |
| hsa04550 | Signaling pathways regulating pluripotency of stem cells - Homo sapiens (human) |
| hsa04150 | mTOR signaling pathway - Homo sapiens (human) |
| hsa05225 | Hepatocellular carcinoma - Homo sapiens (human) |
